# The Structural Effect of Electrode Mesh on Hydrogen Evolution Reaction Performance for Alkaline Water Electrolysis

**DOI:** 10.3389/fchem.2021.787787

**Published:** 2021-11-19

**Authors:** Hae In Lee, Hyun-Seok Cho, MinJoong Kim, Jae Hun Lee, ChangSoo Lee, Sechan Lee, Sang-Kyung Kim, Chang-Hee Kim, Kwang Bok Yi, Won-Chul Cho

**Affiliations:** ^1^ Hydrogen Research Department, Korea Institute of Energy Research (KIER), Daejeon, South Korea; ^2^ Graduate School of Energy Science and Technology, Chungnam National University (CNU), Daejeon, South Korea; ^3^ Department of Advanced Energy and Technology, Korea University of Science and Technology (UST), Daejeon, South Korea; ^4^ Department of Hydrogen Energy, Korea Institute of Energy Technology (KENTECH), Naju-si, South Korea; ^5^ Department of Chemical Engineering Education, Chungnam National University (CNU), Daejeon, South Korea

**Keywords:** alkaline water electrolysis, hydrogen evolution reaction, electrode, complex structured material, nickel woven mesh, nickel expanded mesh

## Abstract

Alkaline water electrolysis (AWE) is a mature water electrolysis technology that can produce green hydrogen most economically. This is mainly attributed to the use of Ni-based materials that are easy to process and inexpensive. The nickel-based meshes with various structures such as woven mesh and expanded mesh are widely used as electrode in the AWE due to its common availability and easy fabrication. However, the morphological effect of meshes on hydrogen evolution reaction (HER) performance has not been studied. Here a new parameter to determine the structural effect of mesh on HER performance was first proposed. The key factors of the parameter were found to be the strand width, pore width and the strand surface area. The woven mesh with the ratio of pore width to strand width that converges to 1 showed the lowest the overpotential. The expanded mesh with the higher the structural surface area exhibited the lowest the overpotential. This study will help to choose an optimal structure for the mesh with the HER electrode.

## Introduction

The world is expanding the supply of renewable energy in order to reduce carbon dioxide emissions. (Shen et al., 2020; Vakulchuk et al., 2020; Wang et al., 2020). Water electrolysis technology is receiving a lot of attention as a key technology that can solve the problem of intermittent renewable energy power generation. (Qadrdan et al., 2015; Ju et al., 2018). Alkaline water electrolysis is an electrochemical technology with a long history that can produce eco-friendly hydrogen (Estermann et al., 2016; Eveloy and Gebreegziabher., 2018) and is one of the easiest methods for hydrogen production among water electrolysis technologies (Zeng and Zhang., 2010; Olivier et al., 2017). Especially, alkaline water electrolysis has the advantage of high economic efficiency as it does not use a noble metal catalyst as an electrode (Speckmann et al., 2019; Jang et al., 2021). A typical material used as an electrode for alkaline water electrolysis is nickel with high intrinsic activity (Zhang et al., 2015; Wang et al., 2017). The cathode, where hydrogen evolution reaction occurs, shows a low overpotential with Raney-type nickel electrode which can increase the electrochemically active surface area by increasing the roughness of the nickel surface (Choquette et al., 1990; Gannon and Dunnill, 2019). Raney-type nickel can be produced simply by depositing elements such as aluminium on the surface of the substrate by co-electroplating or co-sputtering method (Han et al., 2021). Typical materials used as electrode are nickel and stainless steel. In the case of stainless steel, a passivation layer composed of Cr oxide is formed on the surface (Silva et al., 2006), making it difficult to deposit, whereas the Ni-based electrode can be easily stabilized with simple heat treatment after deposition.

**SCHEME 1:**
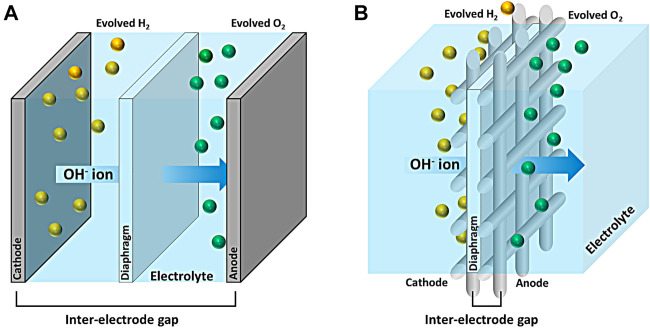
**(A)** Conventional and **(B)** zero-gap design alkaline water electrolytic unit cell.

In regard to the efficiency, the resistance occurs mainly in the electrode, separator, and structure of the cell. There is a gap between the cathode and the anode as shown in Scheme 1A in the traditional water electrolysis cell. The inter-electrode gap leads the distance of hydroxide ion as electrolyte, resulting in a significant increase in ohmic resistance. A zero-gap design cell in Scheme 1B minimized the ohmic resistance by using an electrode with complex structures, which has high surface area with active sites and enough pores to release the entrapped bubble (Nidola, 1984). Currently, most alkaline water electrolysis adopts a zero-gap design cell (Haverkort and Rajaei., 2021;de Groot and Vreman., 2021) equipped with the electrodes in form of mesh (Zayat et al., 2021), foam (Lee et al., 2020), and perforated plate (Jiang et al., 2020). Metal mesh has the advantage of being able to easily manufacture a desired shape.

Woven mesh has two distinct sets of yarns or threads interlaced at right angles. Woven mesh has the advantage of being able to easily determine the thickness of the line and the width of the pores according to the purpose. In general, metal woven mesh is used in various industries such as fences, filters, and electrodes. Metallic expanded mesh is fabricated by pressing the metal plate using a press head having shape of zig zag. The strand width, pore width and shape of expanded metal was determined by the shape and structure of the press head. Expanded metal is used in various fields as a support for buildings and structures. The metallic mesh is widely used as an electrode in alkaline water electrolysis. The effect of the structural properties of metal woven mesh on the hydrogen evolution reaction (HER) in a microbial electrolysis cell has been studied with respect to current density and the size of the hydrogen bubble (Zhang et al., 2010). However, the effect of structural properties of the electrode mesh on the HER in the alkaline water electrolysis have not been studied yet.

In this study, new parameters to understand the effect of structural characteristics of woven mesh and expanded mesh on HER was identified. The structural characteristics of the mesh were identified through the 3-dimetional geometric area and the 2-dimetional coverage area. And the effect of the ratio of pore width to strand width was additionally investigated. The HER performance test was conducted with various structural index.

## Experimental

### Materials

Performance of hydrogen evolution reaction was conducted using 8-types of Ni woven mesh and 5-types of Ni expanded mesh with different wire diameter and pore size. Woven mesh was purchased at NILACO corp (Japan) and expanded mesh was supplied from DEXMET (USA). Woven and expanded mesh was treated in acid (0.1 M HCl solution for 10 min at room temperature) and base solution (1 M NaOH solution for 10 min at room temperature) before hydrogen evolution reaction to remove residual metal powder and organic impurities. The detailed structure parameters of woven mesh and expanded mesh samples was summarized in Tables 1, 2, respectively. Expanded metal sample names in Table 2 were expressed by XNiY-Z, where the X meant nominal thickness (inch), Y represented strand width (inch), and Z indicated the pore width (inch) along the long axis.

**TABLE 1:** Structural parameter of Ni woven mesh samples.

Woven mesh samples	*n,* number of pore ea/cm^2^	*s*, strand width µm	*w,* pore width µm
20 woven mesh	7.87	250	1000
30 woven mesh	11.81	125	700
40 woven mesh	15.75	125	600
50 woven mesh	19.69	125	350
60 woven mesh	23.62	70	350
100 woven mesh	39.37	70	170
150 woven mesh	59.06	50	100
200 woven mesh	78.74	50	70

**TABLE 2:** Structural parameter of Ni expanded mesh samples.

Expanded metal samples	*s*, strand width cm	*t,* strand thickness cm	*w,* pore width cm	*b,* horizontal width cm	*a,* vertical width cm	*m,* number of pore in LWD^a^ ea/cm	*n,* number of pore in SWD^b^ ea/cm
2Ni5-031	0.014	0.01	0.033	0.034	0.024	10.75	17.86
4Ni4-077	0.045	0.01	0.065	0.05	0.035	4.35	8.33
5Ni10-125	0.027	0.012	0.033	0.084	0.043	2.7	5.56
7Ni17-080	0.04	0.008	0.04	0.09	0.06	5.26	7.69
7Ni10-050	0.02	0.008	0.008	0.055	0.04	9.1	17.24

a LWD: Long way of design.

b SWD: Short way of design.

### Electrochemical Active Surface Area

The electrochemical active surface area (ECSA) of the woven mesh and expanded mesh was caculated by cyclic voltammetry (CV) in KOH 30 wt% solution. A Pt plate was used as the counter electrode with Hg/HgO reference electrode. CV is measured at scan rates of 10, 20, 50, and 100 mV/s in the range from −1.0 to −0.1 V. The electrical double layer is calculated in the range from −0.4 to −0.2 V and is the point at which a constant potential occurs due to H-adsorption. The electrochemical active surface area was calculated following equation.

$$ECSA\left(cm^{2}\right)=\frac{S\;\left(V\cdot A\right)/v\;\left(V/s\right)}{Q\left(C/cm^{2}\right)}$$ (1)

where *S* is area of electrical double layer form cyclic voltammetry (V∙A), *v* means scan rate in cycle voltammetry measurements (V/s), and *Q* indicates electrical charge with monolayer adsorption of hydrogen on Ni plate (C/cm^2^).

### Hydrogen Evolution Reaction

Hydrogen evolution reaction was conducted in a half-cell to investigate overpotential in KOH 30 wt% solution as electrolyte. The general three-eclectrode measurement was performed in electrolytic cell. Complexed-structure materials and Pt plate was used as working and counter electrode, respectively, with Hg/HgO reference electrode. The potential required for the hydrogen evolution reaction was supplied by an SP-240 potentiostat (Biologic, France) with EC-lab software. Current was measured from 0 to 300 mA depending on applied potential.

The overpotential is a major factor that can confirm the performance of the electrolytic cell in the hydrogen evolution reacion. Since Hg/HgO/1M KOH electrode is used as the reference electrode, correction is required to calculate the overpotential. The method of calculating the overpotential is as follows.

$$Overpotential\left(V\right)=\left|Applied\;potential\left(V_{Hg/HgO}\right)+0.097+0.059\times \text{pH}\right|$$ (2)

The potential for obtaining a current of 150 mA was compared in this study. The overpotential at 150 mA current of each complex-structured material was calculated and shown in Table 3.

**TABLE 3:** The applied potential and overpotential at 150 mA current of complex-structured material.

Woven mesh samples	Applied potential V_Hg/HgO_	Overpotential mV
20	−1.313	390
30	−1.335	412
40	−1.323	400
50	−1.297	374
60	−1.323	400
100	−1.284	361
150	−1.292	369
200	−1.270	347
Expanded mesh samples		
2Ni5-031	−1.250	327
4Ni4-077	−1.245	322
5Ni10-125	−1.299	376
7Ni17-080	−1.273	350
7Ni10-050	−1.275	352

## Results and Discussion

### Complex-Structured Materials

**FIGURE 1:**
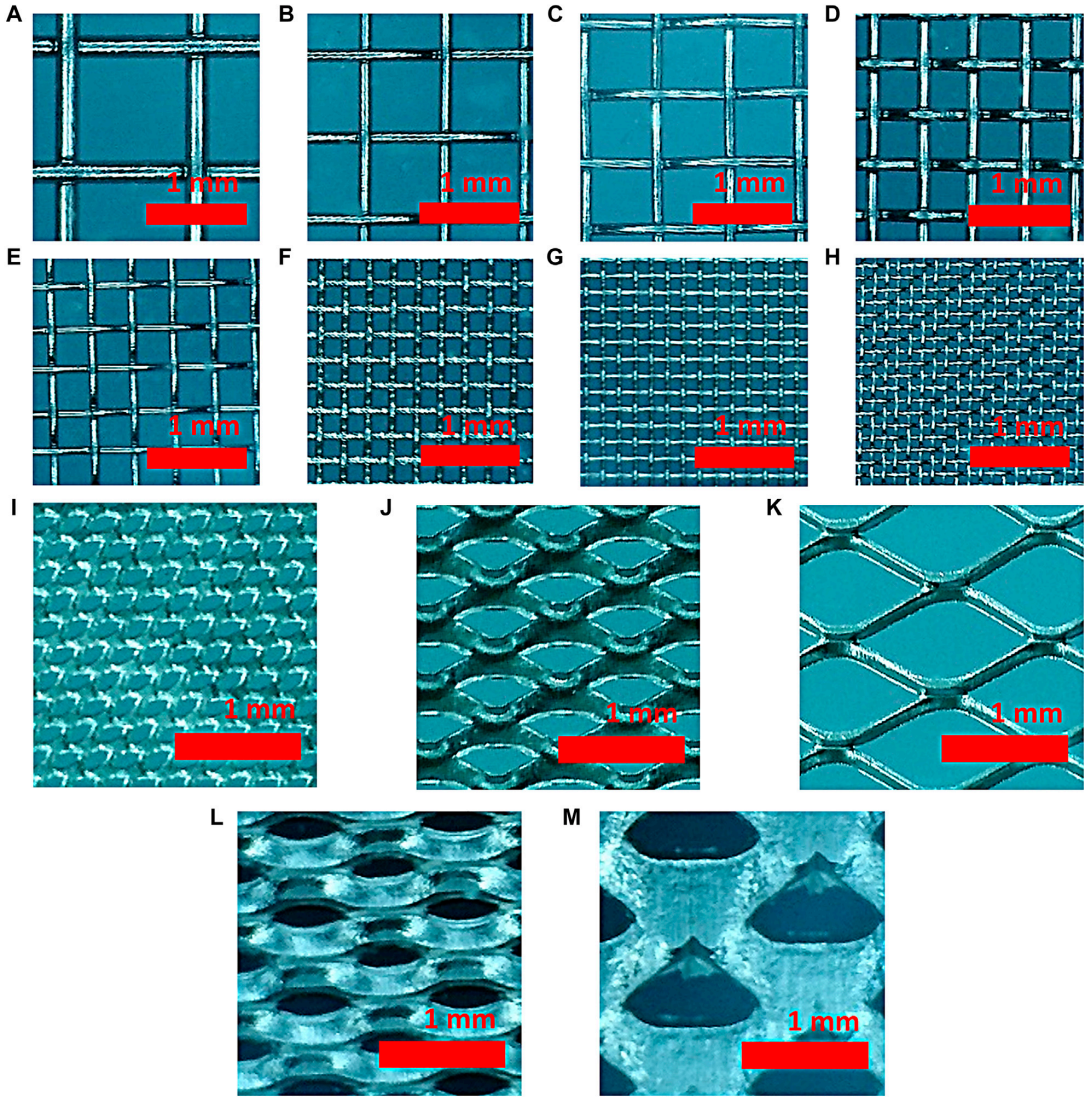
Optical microscopic image of **(A)** 20, **(B)** 30, **(C)** 40, **(D)** 50, **(E)** 60, **(F)** 100, **(G)** 150, and **(H)** 200 woven mesh and **(I)** 2Ni-031, **(J)** 4Ni4-077, **(K)** 5Ni10-125, **(L)** 7Ni10-050, and **(M)** 7Ni17-080 expanded mesh samples.

Eight types of meshes and five types of expanded meshes were prepared to determine the correlation between the performance of hydrogen evolution reaction and the structural properties of the mesh. Figure 1 is a light microscopic image of nickel woven and expanded mesh taken with a light microscope.

Detailed structural parameters of the woven mesh are shown in Table 1. The woven mesh with the largest strand width and pore width is 20 woven mesh, and the woven mesh with the smallest strand width and pore width is 200 woven mesh. The 30, 40, and 50 woven meshes have the same strand width, but the pore widths are progressively smaller, meaning that they become denser. 60, 100 woven mesh and 150, 200 woven mesh have the same strand width at 70 and 50 μm, respectively.

Table 2 shows the structure parameters for the expanded mesh (Figures 1H,I), which shows the irregular strand width and random pore width unlike woven mesh. Thus, it is hardly possible to analyze the structure of the mesh.

### Area Calculation Method

The new parameter to determine the structural surface area of the material was derived in order to check the relationship between the structural properties of complex structural materials and the overpotential in alkaline water electrolysis. First, the structure of woven mesh was calculated using the method reported in the paper of Zhang et al. (2010).

**SCHEME 2:**
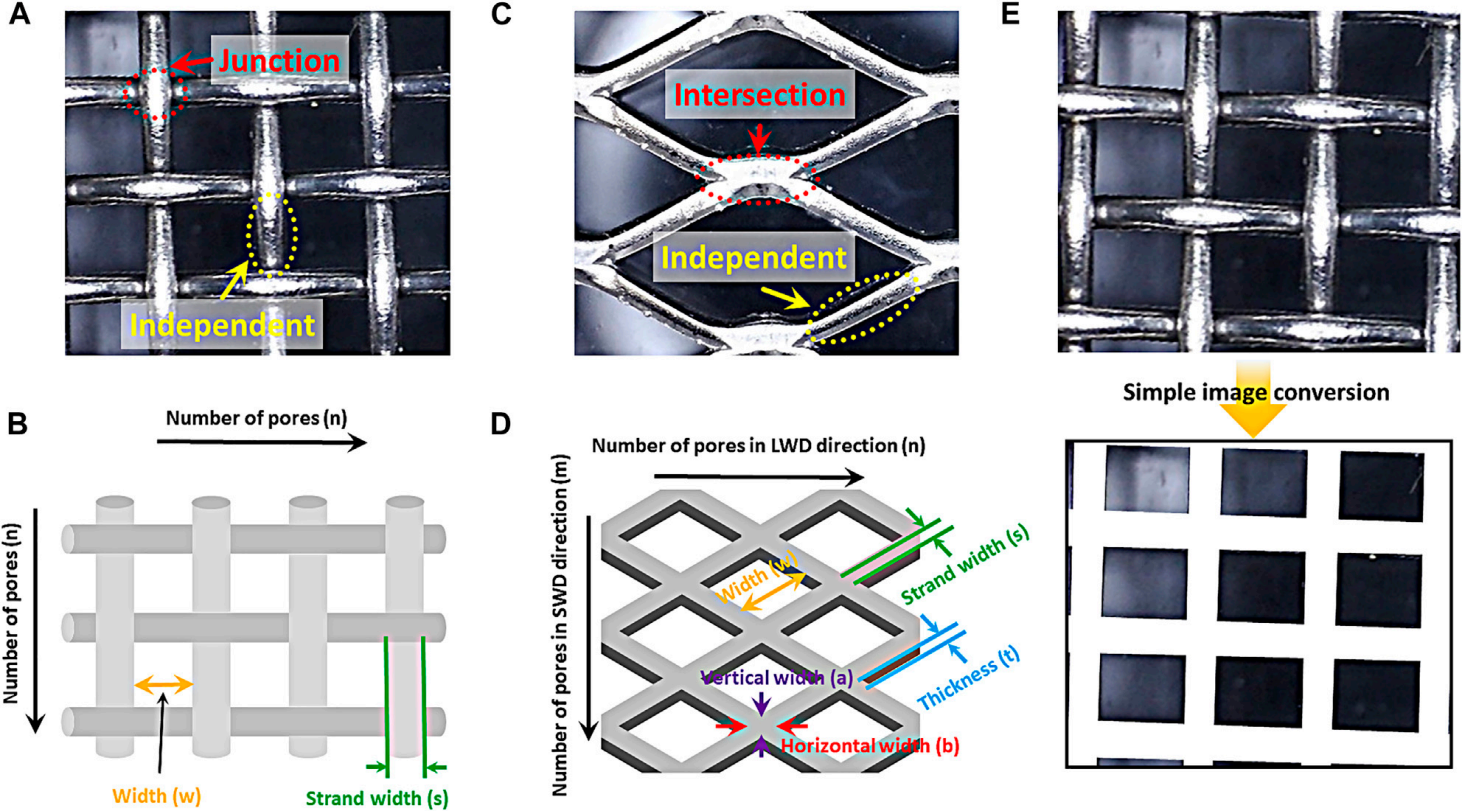
Optical images of **(A)** woven mesh sample and **(C)** expanded mesh sample. Schematic diagram of **(B)** woven mesh sample and **(D)** expanded mesh sample. **(E)** Optical image and the image conversion of the coverage area of woven mesh sample.

The woven mesh is composed of a junction segments and an independent segments as shown in Scheme 2A. The independent segment is the shape of a cylinder, and the structure surface area can be calculated as follows;

$$Area\;of\;independent\;segment\;\;\left(cm^{2}\right)\;=\pi \times \frac{1}{2}\times s\;\left(cm\right)\times w\;\left(cm\right)$$ (3)

where *s* and *w* is strand and pore width (cm), respectively. And the area of the junction can be calculated by the following formula.

$$Area\;of\;juntion\;segment\;\left(cm^{2}\right)\;=\frac{3}{2}\times \pi \times \frac{1}{4}\times s^{2}\;\left(cm\right)$$ (4)

The number of independent and junction segment (Scheme 2B) can be calculated using the number of pores (*n*). The number of independent segments is *2n*(*n+1*), and (*n+1*)^*2*^ is the number of junction segments. The structural surface area of the woven mesh can be calculated by calculating the area of the independent and junction segments and multiplying each number.

$$Structural\;surface\;area\;of\;woven\;mesh\;\left(-\right)=\left\{2n\left(cm^{-1}\right)\left[n\left(cm^{-1}\right)+1\right]\right\}\times \left[\pi \times \frac{1}{2}\times s\;\left(cm\right)\times w\;\left(cm\right)\right]+\left[{\left(n+1\right)}^{2}\right]\times \left[\frac{3}{2}\times \pi \times \frac{1}{4}\times s^{2}\;\left(cm^{2}\right)\right]$$ (5)

Next, the structural surface area of the expanded mesh was calculated. The expanded mesh is divided into two parts: an independent segment and an intersection segment as shown in Scheme 2C. The shape of the independent part is a cuboid, and the area can be calculated as follows.

$$Area\;of\;independent\;\text{seg}ment\left(cm^{2}\right)\;=2\times \left[s\;\;\left(cm\right)+t\;\;\left(cm\right)\right]\;\times w\;\left(cm\right)$$ (6)

where *s* represents strand width (cm), *t* means thickness (cm), and *w* is pore width (cm). The shape of the intersection segment can be calculated using the following formula as a rhombus.

$$\;Area\;of\;intersection\;\text{seg}ment\;\left(cm^{2}\right)\;=2\times \frac{1}{2}\times a\;\;\left(cm\right)\times b\;\;\left(cm\right)$$ (7)

where *a* and *b* is vertical and horizontal width of rhombus (cm), respectively. The number of intersection and independent segment can be calculated using the number of pores (LWD: *n*, SWD: *m*) in the direction of long way of design (LWD) and short way of design (SWD) as shown in Scheme 2D. The number of non-overlapping pores on each side is equal to the number of pores in the LWD multiplied by the number of pores in the SWD. Structural surface area can be calculated using the following formula.

$$Structural\;surface\;area\;of\;expanded\;mesh\;\left(-\right)=n\left(cm^{-1}\right)\times m\left(cm^{-1}\right)\times \left[8\times s\;\left(cm\right)\times w\;\left(cm\right)+8\times t\;\left(cm\right)\times w\;\left(cm\right)+2\times a\;\left(cm\right)\times b\;\left(cm\right)\right]$$ (8)

The coverage area, which is a 2-dimentional factor, is shown in Scheme 2E and it was calculated using the simple image conversion method, which calculates the 2-dimentional area of a structure via an optical image without considering a special measurement or calculation formula. The area occupied by strand among images of complex electrode photographed through the simple image conversion method was converted into a single color (white). The area ratio of the single color part occupied by the strand in the total area of the converted image is defined as the coverage area.

$$Coverage\;area\;\left(\%\right)=\frac{Area\;of\;white\;color\;\left(cm^{2}\right)}{Total\;area\;\left(cm\right)}\times 100\left(\%\right)$$ (9)

The calculated structural surface area and coverage area of woven mesh and expanded mesh are shown in Tables 4, 5, respectively. The structural surface area was calculated according to the use of a circular electrode with a diameter of 2 cm.

**TABLE 4:** Characteristic of Ni woven mesh.

	Structural surface area (cm^2^)	Coverage area (%)	Ratio of pore width to strand width (-)
20 woven mesh	2.379	37	4.0
30 woven mesh	1.770	35	5.6
40 woven mesh	2.396	28	4.8
50 woven mesh	1.944	57	2.8
60 woven mesh	1.628	40	5.0
100 woven mesh	1.951	56	2.4
150 woven mesh	1.793	59	2.0
200 woven mesh	2.196	74	1.4

**TABLE 5:** Characteristic of Ni expanded mesh.

	Structural surface area (cm^2^)	Coverage area (%)
2Ni5-031	2.402	61.95
4Ni4-077	1.826	69.38
5Ni10-125	0.413	46.00
7Ni17-080	1.661	75.73
7Ni10-050	1.525	85.22

The manufacturing parameters are strand width and pore width in the manufacturing method of woven mesh. Thus, the ratio of pore width to strand width can also be considered as a structural property. The ratio of pore width to strand width was calculated using the following formula.

$$Ratio\;of\;Pore\;to\;strand\;width\;\left(\%\right)=\frac{Pore\;width\;\left(cm\right)}{Strand\;width\;\left(cm\right)}\times 100\;\left(\%\right)$$ (10)

The ratio of pore width to strand width can be considered as a 1-dimensional structural parameter because it is a comparison between a line (pore width) and a line (strand width). The calculated result of the ratio of pore width to strand width is shown in Table 4.

### Relationship of HER With Structural Properties of Mesh

**FIGURE 2:**
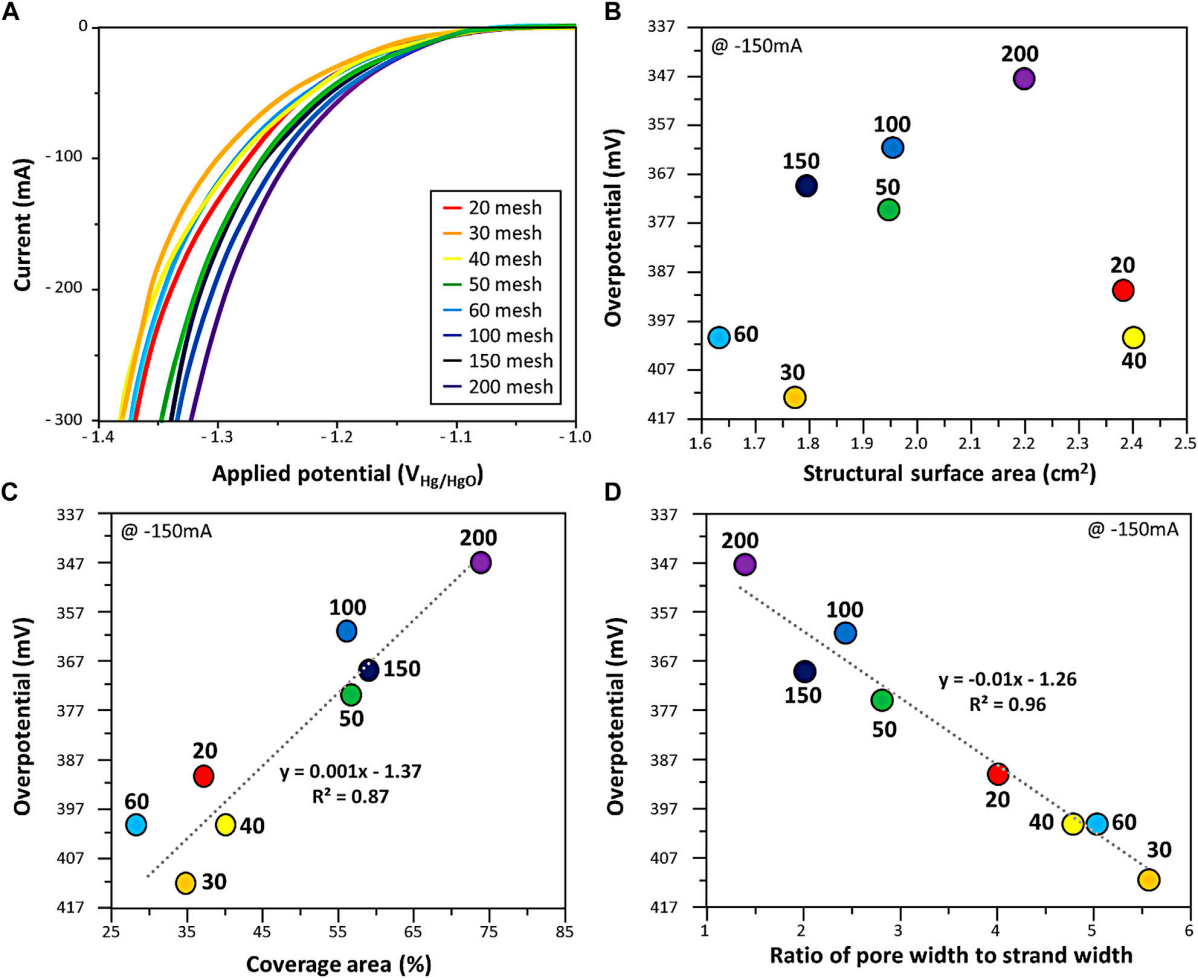
**(A)** HER performance of woven mesh. The overpotential at 150 mA current in HER with respect to **(B)** structural surface area, **(C)** coverage area, and **(D)** ratio of pore width to strand width of woven mesh. The dotted line is the linear regression plot obtained from the data.

Hydrogen evolution reaction was performed using eight types of Ni woven mesh as cathode (Figure 2A). Figure 2B is the overpotential at 150 mA current according to the structural surface area, which is a 3-dimentional factor. There was little relationship between the overpotential and the structural surface area.

The coverage area, 2-dimentional factor and overpotential at 150 mA current were correlated in Figure 2C. The coverage area of the woven mesh lower the overpotential in the HER. The effect of the ratio of pore width to strand width was investigated in Figure 2D. The overpotential decrease as the ratio of pore width to strand width converges to 1. It was found that the ratio of pore width to strand width, the 1-dimensional factor has a higher tendency to overpotential than the structural surface area, 3-dimensional factor.

The increase in the coverage area, 2-dimensional area of the working electrode facing the counter electrode, is sufficient for HER. In addition, the ratio of pore width to strand width of woven mesh exhibited low overpotential when mesh strands and pores were uniformly arranged.

**FIGURE 3:**
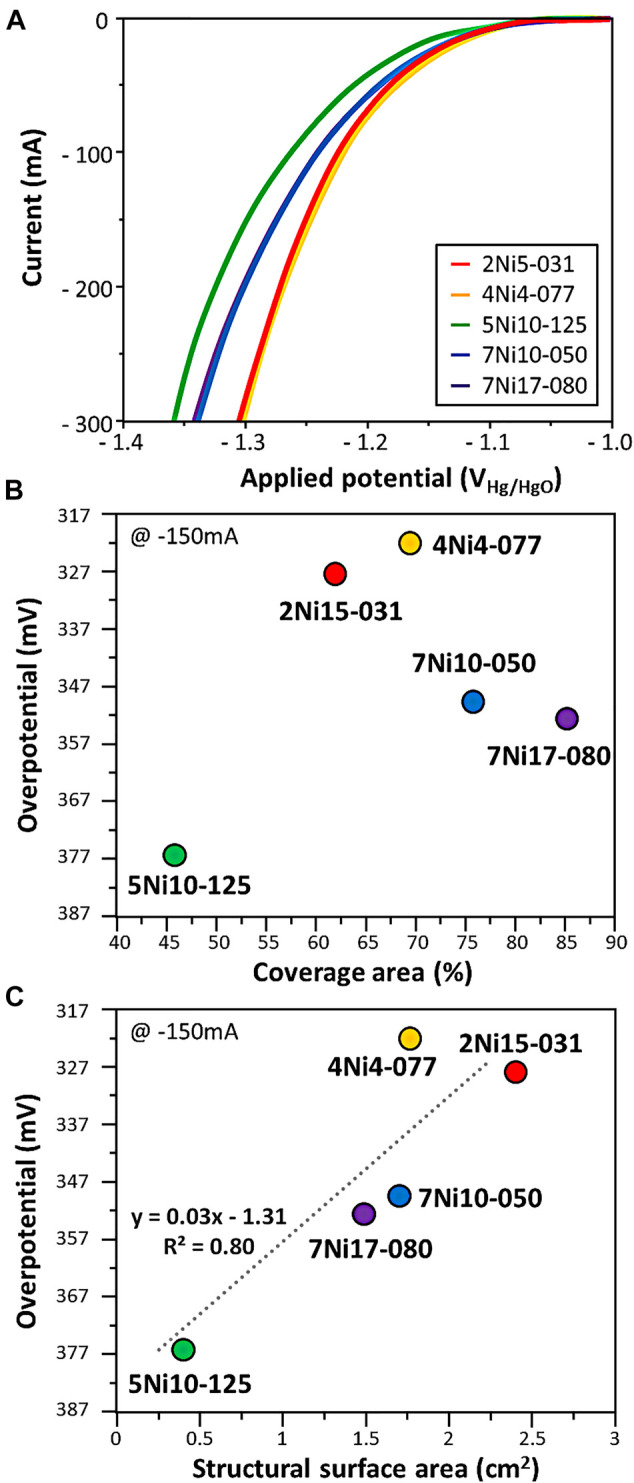
**(A)** HER performance of expanded mesh. The overpotential at 150 mA current in HER with respect to **(B)** coverage area and **(C)** structural surface area. The dotted line is the linear regression plot obtained from the data.

Figure 3A shows the measured current according to the overpotential in the hydrogen evolution reaction of the expanded mesh. The overpotential of the four expanded meshes increased as the coverage area increased. The structural surface area, which is a 3-dimentional factor, showed low overpotential. As the structural surface area increased from 0.41 to 2.40 cm^2^, the overpotential decreased from 376 to 322 mV. From the relationship between overpotential and structural properties in the expanded mesh, it seemed that the structural surface area, which is a 3-dimentional factor, has a greater effect than the coverage area, which is a 2-dimentional factor. The ratio of pore width to strand width, a 1-dimentional factor (Figure 2D) considered in the woven mesh, was excluded due to the characteristics of the expanded mesh having an irregular pore structure.

**FIGURE 4:**
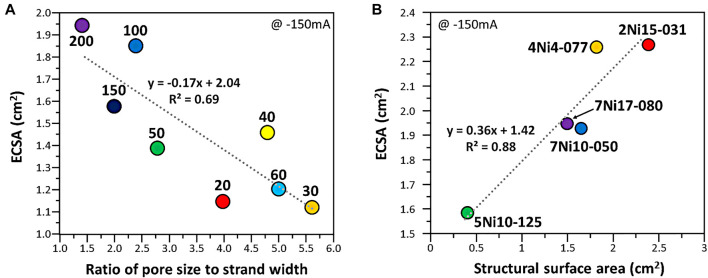
Electrochemically active surface area values as a function of **(A)** ratio of pore width to strand width of woven mesh and **(B)** structural surface area of expanded mesh. The dotted line represents the linear regression plot obtained from the data.

The higher value of electrochemically active surface area (ECSA) shows high performance in an electrochemical reaction. ECSA of woven mesh and expanded mesh were measured and the structural characteristics were compared. The ratio of pore width to strand width of the woven mesh was compared with the electrochemically active surface area (Figure 4A). As the ratio of pore width to strand width converges to 1, it showed a high electrochemically active surface area. And the relationship between the electrochemically active surface area of the expanded mesh and the structural surface area was tested (Figure 4B). The electrochemically active surface area increased as the structural area increased for the expanded mesh, which is in line with the previous study results (Figure 3C).

## Conclusion

We studied that the structural characteristics of the woven mesh and expanded mesh as a cathode electrode for alkaline water electrolysis. The junction segments and independent segments were important structure for the woven mesh, while the intersection segments and independent segments was considered in the expanded mesh. The ratio of pore width to strand width, 1-dimensional factor, affects the overpotential of the HER in the woven mesh. Meanwhile, the structural surface area, 3-dimentional factor, had a greater effect on the expanded mesh. These results was confirmed by the electrochemically active surface area measurement. This result will help to select the electrode structure in alkaline water electrolysis.

## Data Availability

The original contributions presented in the study are included in the article/Supplementary Material, further inquiries can be directed to the corresponding authors.

## Nomenclature

*a*: Vertical width of rhombus (cm)
*b*: Horizontal width of rhombus (cm)
*n*: Number of pore (ea)
*Q*: Electrical charge (C/cm^2^)
*S*: Area of electrical double layer (V∙A)
*s*: Strand width (cm)
*t*: Thickness (cm)
*v*: Scan rate (V/s)
*w*: Pore width (cm)
AWE: Alkaline water electrolysis
ECSA: Electrochemically active surface area
HER: Hydrogen evolution reaction
LWD: Long way of design
SWD: Short way of design
